# Anti-claudin-4 extracellular domain antibody enhances the antitumoral effects of chemotherapeutic and antibody drugs in colorectal cancer

**DOI:** 10.18632/oncotarget.26427

**Published:** 2018-12-21

**Authors:** Rina Fujiwara-Tani, Takamitsu Sasaki, Yi Luo, Kei Goto, Isao Kawahara, Yukiko Nishiguchi, Shingo Kishi, Shiori Mori, Hitoshi Ohmori, Masuo Kondoh, Hiroki Kuniyasu

**Affiliations:** ^1^ Department of Molecular Pathology, Nara Medical University, Kashihara, Nara 634-8521, Japan; ^2^ Jiangsu Province Key Laboratory of Neuroregeneration, Nantong University, Nantong, Jiangsu 226001, China; ^3^ Drug Innovation Center, Graduate School of Pharmaceutical Sciences, Osaka University, Suita, Osaka 565-0871, Japan

**Keywords:** claudin, tight junction

## Abstract

Claudin-4 (CLDN4) is a major epithelial tight junction protein overexpressed in many cancers to maintain the tumor environment. In this report, we aimed to determine the efficacy of targeting CLDN4 in colorectal cancer (CRC) using an anti-CLDN4 extracellular domain antibody, 4D3. CLDN4 was upregulated in CRC metastatic foci. CLDN4 expression in CRC cells was reduced by upregulation of TNFα, which was induced by *Clostridium perfringens* enterotoxin produced by gut flora. In a nude mouse liver metastasis model, inhibition of metastasis was increased by combination treatment with 5-fluorouracil (FU) and 4D3 compared to that with 5-FU alone. Moreover, combination treatment with 4D3 and anti-epithelial growth factor receptor (EGFR) antibody C225 resulted in more pronounced inhibition of *in vitro* sphere formation and tumor growth in nude mice compared to that observed with C225 alone. Moreover, the time interval between the administration of 4D3 and that of C225 was important for maximizing the C225-induced inhibition of EGFR phosphorylation. In a nude mouse model, sequential treatment with 4D3 and C225 with a 6-h time interval resulted in more pronounced inhibition of tumor growth than concurrent treatment. These findings suggest that the targeting of CLDN4 enhances the antitumoral effects of chemotherapeutic agents and molecular targeting antibodies when used in combination.

## INTRODUCTION

Colorectal cancer (CRC) is the third leading cause of cancer-related deaths in Japan, and in the last four decades, its incidence has risen sharply [1]. This is in part due to the increase in Western lifestyles and, in particular, high-fat and high-glucose diets. In spite of advances in treatment, those with metastatic disease still have a poor prognosis. One-fourth of advanced cases are associated with liver metastasis, which is a life-threatening event accounting for 30% of CRC deaths [2, 3]. However, there are indications that prompt adjuvant chemotherapy upon evidence of liver metastasis may improve patient prognosis.

Claudins (CLDN) are the major protein components of tight junctions, which seal adjacent cells together at adherens junctions and desmosomes [4]. Twenty-seven different but highly related CLDN family members have been identified [5]. The precise combination of CLDN proteins expressed in cells is dependent on location and cell type [6]. Tight junctions play an essential role in the establishment and maintenance of cell polarity and are involved in the regulation of other cellular functions, including proliferation and differentiation. Tight junctions prevent the lateral permeation of lipids and membrane proteins containing signaling molecules [7]. The border formed by a tight junction thus maintains the differential compositions of the apical and basolateral regions [5, 7].

CLDN4 is commonly expressed in the mucosal epithelium and epithelial tumors. Expression of CLDN4 is upregulated in several types of epithelial malignancies, including pancreatic, urinary bladder and ovarian cancer [8–14]. CLDN4 expression is also associated with malignant phenotypes, including metastasis [6, 15, 16]. Ovarian cancer cells expressing CLDN4 show increased invasion, motility, and anti-apoptotic survival [17], and CLDN4 overexpression has been observed in advanced ovarian cancer [9]. CLDN4 expression has also been associated with a more invasive phenotype in pancreatic intraductal papillary mucinous neoplasms [14]. Thus, CLDN4 is a relevant molecular target for the treatment of epithelial cancers and may be inhibited by drugs including *Clostridium perfringens* enterotoxin (CPE) and antibodies [15, 16, 18, 19]. We recently developed a monoclonal antibody specific to the extracellular domain of CLDN4, named 4D3, which exerts antitumoral effects in bladder cancer models [11].

In the present study, we aimed to determine whether this antibody is also an effective suppressor of metastasis in CRC. Our results provide new insight into the antitumoral effects of 4D3 and have potential relevance for the treatment of patients with CRC.

## RESULTS

### Expression of CLDN4 in CRC

**Figure 1:**
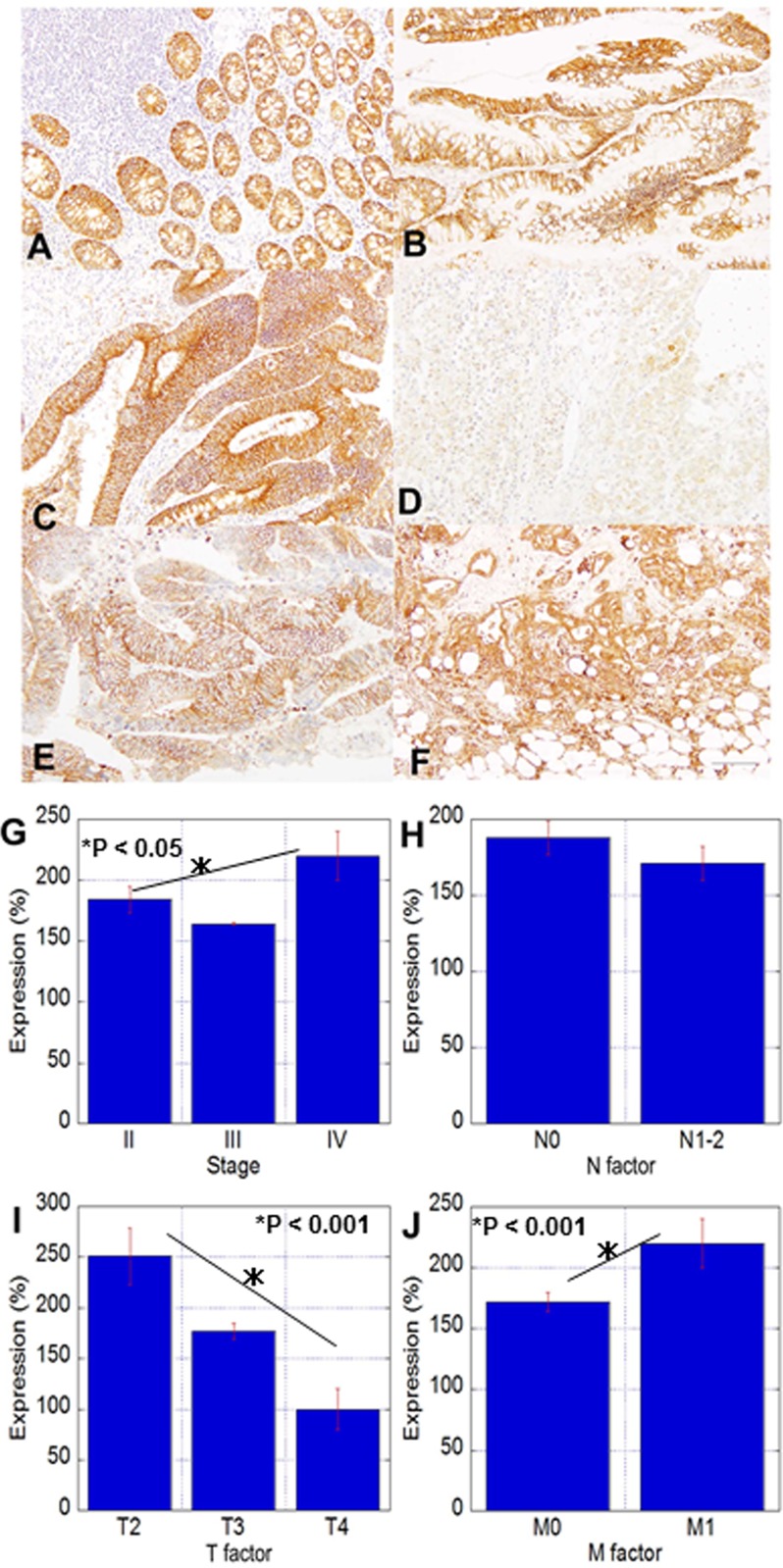
CLDN4 expression and correlations with clinicopathological parameters in CRC (**A**–**E**) Immunohistochemistry for CLDN4 in colonic mucosa and tumors; A: non-tumoral colonic mucosa, B: adenoma, C: adenocarcinoma, pT3, pN0, D: adenocarcinoma, pT3, pN0, H2, P1, E: liver metastasis of Case D, (**F**) peritoneal metastasis of Case D. Bar, 100 μm. (**G**–**J**) Relationship between CLDN4 expression and clinicopathological parameters. G: pathological stage, H: nodal metastasis, I: tumor expansion, J: distant metastasis. The expression index 100 is the expression level in the normal epithelium. Error bars, SD.

Expression of CLDN4 was examined in 124 cases of CRC (Figure 1 and Table 1). CLDN4 was expressed on the cytoplasmic membrane in normal colonic epithelial and cancer cells (Figure 1A–1C). In the most of cases we examined, intratumoral heterogeneity of CLDN4 was detected. Paradoxically, in the case shown in Figure 1D–1F, the primary tumor showed faint CLDN4 expression, whereas metastatic sites in the liver and peritoneum showed strong expression. This upregulation of CLDN4 in distant metastatic sites was found in 13 of 14 stage IV cases.

**Table 1:** Examined CRC cases

Age	67 yo (42 – 93 yo)		
Sex	Male: 51	Female: 63	
pT	pT2: 4 cases	pT3: 108 cases	pT4: 2 cases
pN	pN0: 47 cases	pN1: 65 cases	pN2: 2 cases
pM	pM0: 100 cases	pM1: 14 cases	
pStage	II: 43 cases	III: 57 cases	IV: 14 cases
Grade	G1: 22 cases	G2: 72 cases	G3: 20 cases

1. Clinicopathological classification is according to UICC-TNM Classification [40]. Pathological T-factor (pT): pT2 (tumor invades muscularis propria), pT3 (tumor invades through the muscularis propria into the pericolorectal tissues), and pT4 (tumor penetrates to the surface of the visceral peritoneum or invades other organs).

2. Pathological N-factor (pN): pN0 (no regional lymph node metastasis), pN1 (metastasis in 1-3 regional lymph nodes), pN2 (metastasis in 4 or more lymph nodes).

3. Pathological M-factor (pM): pM0 (no distant metastasis), pM1(distant metastasis).

4. Pathological stage: stage II (cases invaded into the muscularis propria layer or above), stage III (any cases with lymph node metastasis), and stage IV (any case with or without lymph node metastases but with distant metastases; 8 liver metastasis cases, 3 peritoneal metastasis cases, 3 lung metastasis cases).

5. Histological grade (Grade) is according to UICC-TNM Classification (Gospodarowicz *et al*, 2009). G1 (well-differentiated), G2 (moderately differentiated), G3 (poorly differentiated).

Analysis of CLDN4 expression and clinicopathological parameters showed that CLDN4 levels were inversely correlated with T factor; in contrast, CLDN4 expression was positively correlated with distant metastasis and pathological stage (Figure 1G–1J).

### Expression of CLDN4 in human CRC cell lines

**Figure 2:**
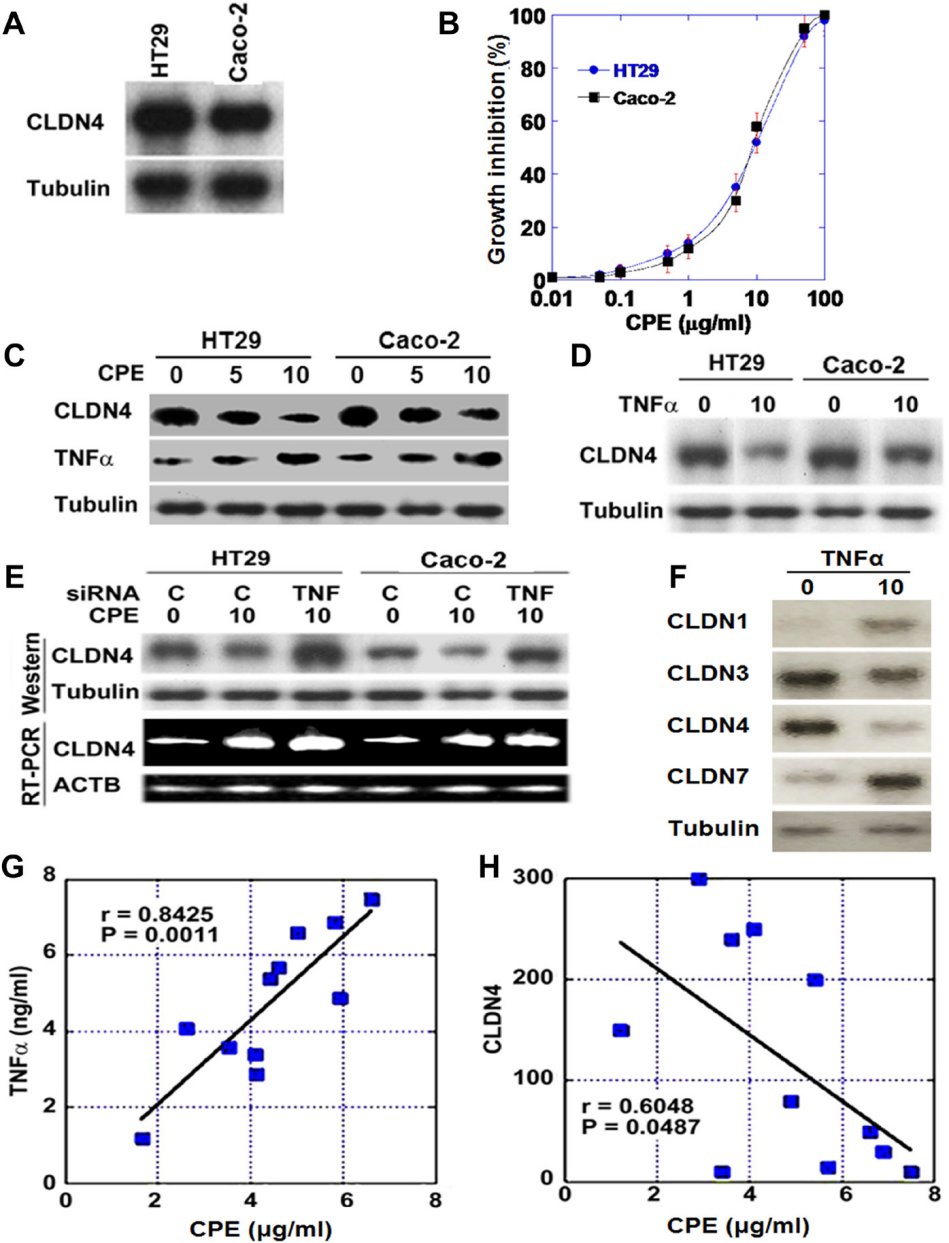
Effect of *Clostridium perfringens* enterotoxin (CPE) on CLDN4 expression (**A**) Expression of CLDN4 protein in HT29 and Caco-2 human CRC cells. (**B**) Growth inhibitory effect of CPE in CRC cells. (**C**) Expression of CLDN4 and TNFα in CPE-treated CRC cells. (**D**) Expression of CLDN4 in TNFα-treated CRC cells. (**E**) Effect of knockdown of TNFα on CLDN4 expression in CPE-treated CRC cells. (**F**) Effect of TNFα on protein levels of epithelial claudins was examined. (**G**–**H**) Relationship between concentrations of CPE and TNFα (G) or between concentrations of CPE and CLDN4 (H). Concentrations of CPE and TNFα were measured by ELISA in the fresh-frozen tumor tissues of 11 human CRCs. Tubulin or *ACTB* served as loading controls.

Human CRC cell lines HT29 and Caco-2 were examined for CLDN4 expression (Figure 2A). Levels were higher in HT29 cells than in Caco-2 cells. We also examined the effect of CPE, which is produced by commensal bacteria in the colonic mucosa, on cell proliferation (Figure 2B). Cell proliferation of CRC cell lines was inhibited in a dose-dependent manner. As shown in Figure 2C, CPE treatment increased TNFα and decreased CLDN4 expression in both CRC cell lines. Direct treatment with TNFα also decreased CLDN4 expression (Figure 2D). Interestingly, CPE treatment increased levels of *CLDN4* mRNA but decreased levels of the corresponding protein (Figure 2E). In contrast, following the silencing of TNFα with siRNA, CPE treatment increased both the mRNA and protein levels of CLDN4. We examined also effect of TNFα on other epithelial claudins (Figure 2F). Protein levels of CLDN3 and CLDN4 were decreased by TNFα, whereas those of CLDN1 and CLDN7 were increased.

### Expression of CLDN4 and TNFα in comparison with CPE in human CRC cases

Given that CLDN4 expression is affected by the levels of TNFα and CPE in the colonic mucosa, we examined the levels of CLDN4 and TNFα and compared them with fecal CPE levels in human CRC cases (Figure 2G and 2H). This revealed linear correlations between levels of CPE and TNFα, whereas it showed inverse correlation between CPE and CLDN4.

### Effect of anti-CLDN4 antibody on human CRC cells

**Figure 3:**
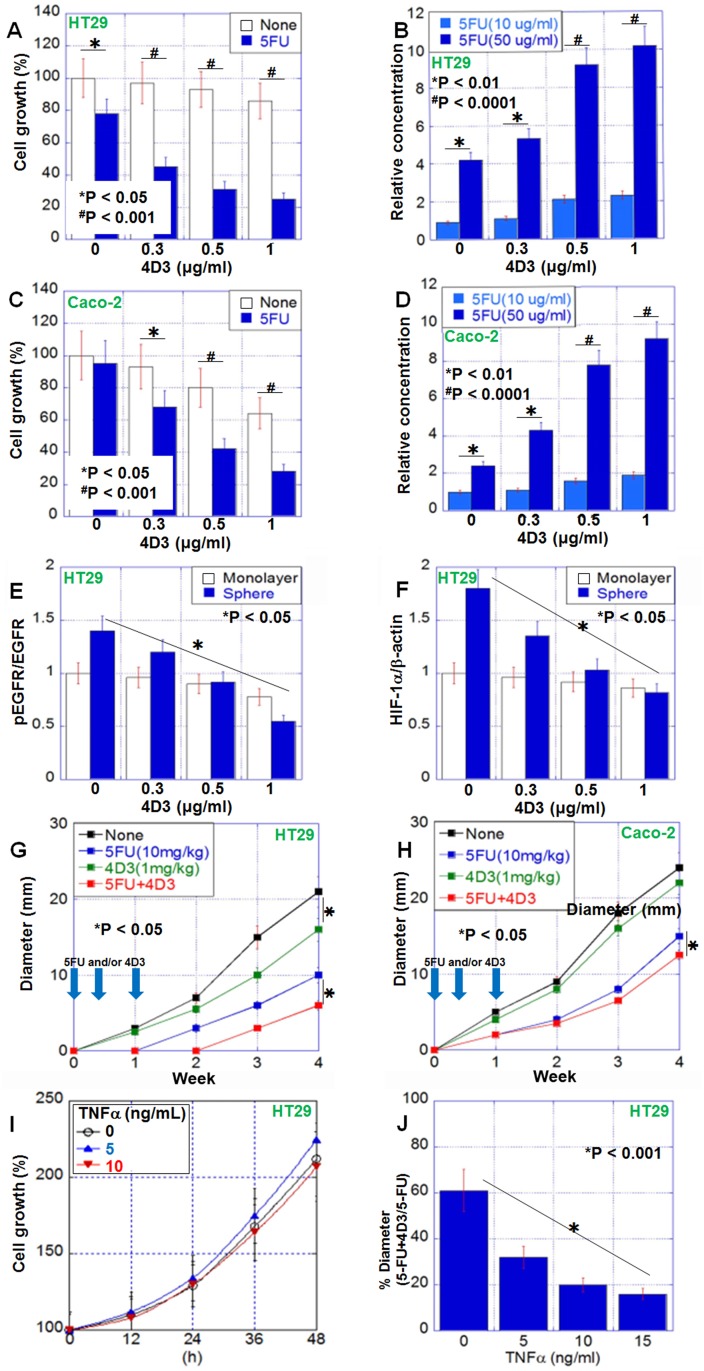
Antitumoral effect of 4D3 anti-CLDN4 antibody in human CRC cells (**A**, **C**) 4D3 enhances the growth inhibitory effects of 5-FU in HT29 cells (A) and Caco-2 cells (C). (**B**, **D**) 4D3 increases the effective intracellular concentration of 5-FU in HT29 cells (B) and Caco-2 cells (D). (**E**, **F**) Effect of 4D3 on EGFR phosphorylation (E) and HIF-1α expression (F) in HT29 cells. (**G**, **H**) Effect of treatment with 5-FU and/or 4D3 without TNFα (G) or with TNFα pretreatment (10 ng/mL, for 2 days) (H). *n* = 5 per group. (**I**) Effect of TNFα on growth of HT29 cells. Graph legend; TNFα (ng/mL). (**J**) Effect of TNFα on the growth inhibition induced by concurrent treatment with 5-FU and 4D3. Error bars, SD.

Next, we examined the effect of 4D3, an anti-CLDN4 extracellular domain antibody, on HT29 and Caco-2 cells with or without concurrent treatment with 5-FU (Figure 3A, 3C). Following treatment with 4D3 antibody alone, no significant growth inhibition was observed. In contrast, the growth inhibitory effect of 5-FU was significantly enhanced by concurrent treatment with 4D3 antibody in a 4D3 dose-dependent manner. The 22% inhibition of growth observed with 5-FU (10 μg/mL) treatment alone was increased to 75% by concurrent treatment with 4D3 (1 μg/mL). Moreover, the intracellular concentration of 5-FU was increased in a 4D3 dose-dependent manner in the two cell lines (Figure 3B, 3D). In HT29 cells treated with 4D3, the ratio of phosphorylated EGFR levels to EGFR levels and HIF-1α expression were decreased in sphere assay but not monolayer assay (Figure 3E, 3F).

The effect of 4D3 was next examined in a mouse model (Figure 3G, 3H). Subcutaneous tumors of HT29 and Caco-2 cells were treated with 5-FU and/or 4D3. Treatment with 4D3 alone, 5-FU alone, and 5-FU plus 4D3 inhibited growth by 16%, 48%, and 68%, respectively, in HT29 cells. In contrast, treatment with 4D3 alone, 5-FU alone, and 5-FU plus 4D3 inhibited growth by 8%, 38%, and 48%, respectively, in Caco-2 cells, which expressed CLDN4 at lower levels than those in HT29 cells. Thus, concurrent treatment with 4D3 provided more pronounced enhancement to growth inhibitory effect of 5-FU in HT29 cells with high levels of CLDN4 than that in Caco-2 cells with low levels of CLDN4. In HT29 cells, TNFα treatment did not affect the cell growth in *in vitro* (Figure 3I). In contrast, the enhanced effects of the combined treatment were abrogated when cells were pretreated with human TNFα (Figure 3J).

### Effect of anti-CLDN4 antibody on metastasis of human CRC cells

**Figure 4:**
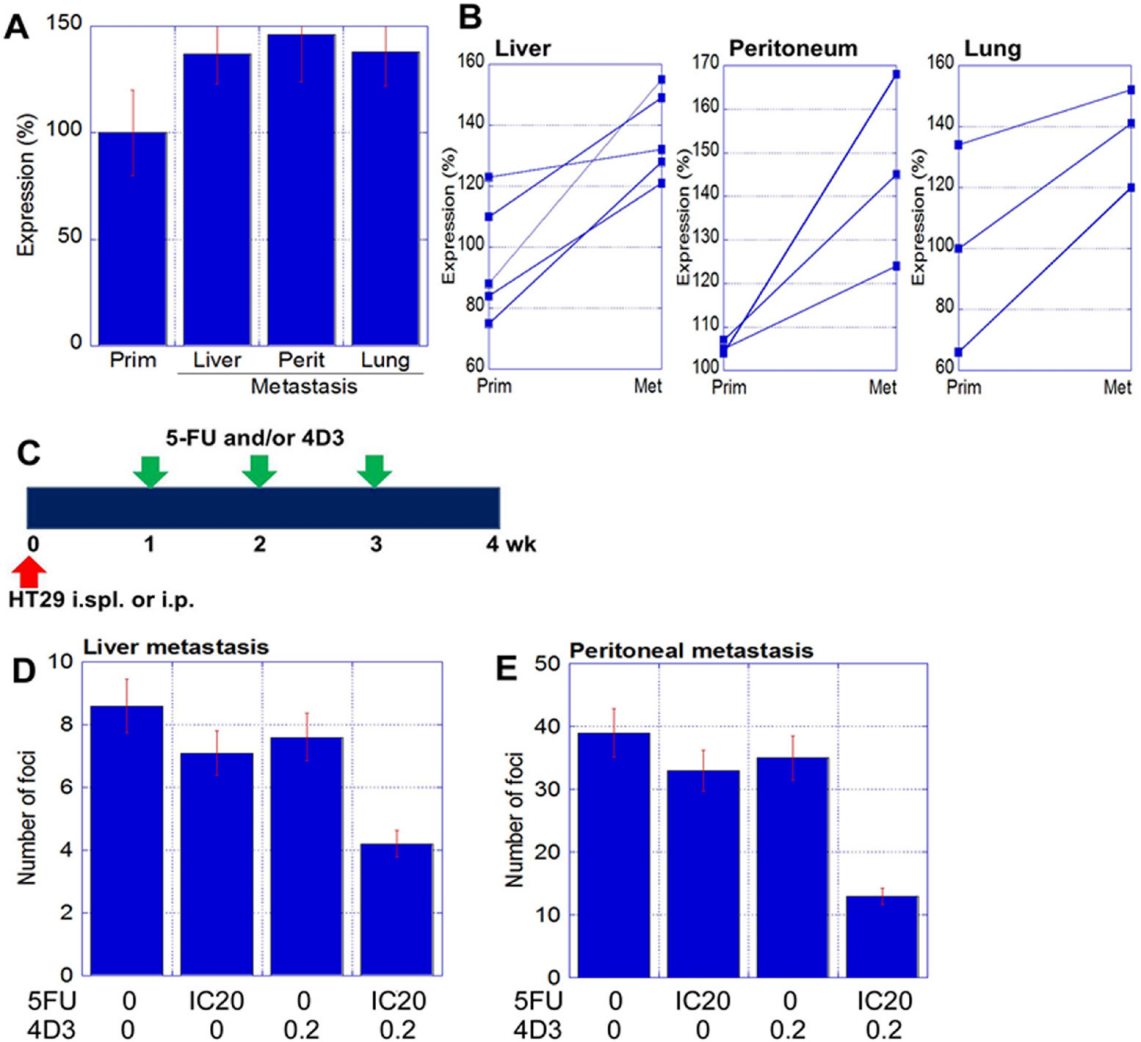
Antitumoral effect of 4D3 anti-CLDN4 antibody in human CRC metastasis (**A**) Expression of CLDN4 in the metastatic foci of 11 CRC cases, as measured by ELISA. (**B**) Alteration of CLDN4 expression in primary tumors (prim) versus metastatic foci (met), with paired samples joined by lines. (**C**) Validation of the synergy between 5-FU and 4D3 in liver and peritoneum metastatic models (*n* = 5 per group). 5-FU (3 mg/kg; equivalent to IC20) and/or 4D3 (0.2 mg/kg) were administrated intraperitoneally. (D and E) Number of metastatic foci in the liver (**D**) and peritoneum (**E**), respectively. Error bars, SD.

As mentioned above, immunohistochemistry of human CRC cases indicated that CLDN4 was expressed at higher levels in the distant metastatic foci than in the primary tumors (Figure 1). We next compared CLDN4 expression among metastasized organs (Figure 4A). According to ELISA examination, CLDN4 protein levels were not significantly different among the liver, peritoneum, and lung. However, CLDN4 levels in metastatic foci were, on average, 1.7 times higher than in primary tumors (Figure 4B).

Next, we examined the effect of 4D3 antibody combined with 5-FU treatment on the formation of metastatic foci (Figure 4C–4E). HT29 cells were inoculated into the spleen and the peritoneum to induce metastasis to the liver and the peritoneum, respectively. Beginning one week after inoculation, established metastatic foci were treated with 4D3 alone, 5-FU alone, or 4D3 plus 5-FU once a week. While 4D3 or 5-FU alone resulted in weak inhibition of tumor growth, concurrent treatment with both agents provided a synergic effect; the growth inhibitory rates of the combination treatment were 5- and 2.3-fold higher in the liver and peritoneum models, respectively, than those elicited by 4D3 or 5-FU alone.

### Effect of 4D3 on antitumoral effect of anti-EGFR antibody C225

**Figure 5:**
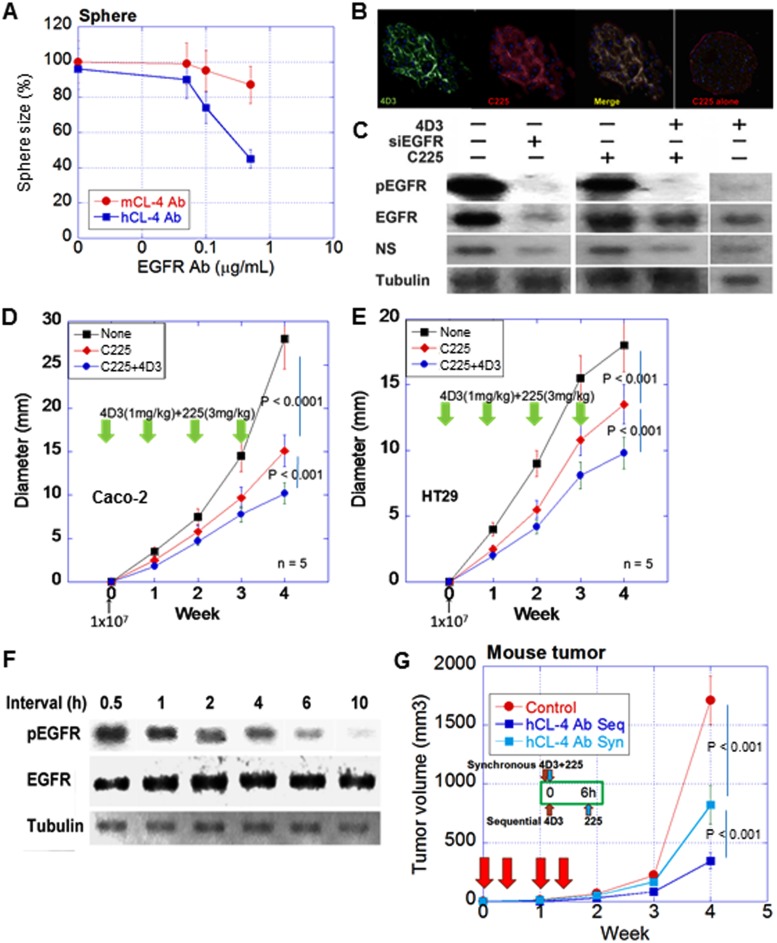
Synergic effect of 4D3 anti-CLDN4 antibody with anti-EGFR antibody (**A**) Effect of 4D3 on growth inhibition induced by anti-EGFR antibody C225 in spheres of Caco-2 cells. Anti-mouse CLDN4 was treated as a negative control. (**B**) Permeation of Caco-2 cell spheres with antibodies. Spheres were treated with fluorescence-labeled 4D3 and C225. Nuclei were stained by DAPI. (**C**) Effect of co-treatment with 4D3 and C225 on phosphorylation of EGFR in Caco-2 cell spheres. (**D**, **E**) Effect of 4D3 on C225-induced inhibition of tumor growth in nude mice (*n* = 5). (**F**) Effect of time interval (h) between treatment with 4D3 and treatment with C225 on phosphorylation of EGFR in spheres. (**G**) Effects of sequential (Seq, 6-h time interval) vs. synchronous (Syn) treatment with 4D3 and C225 on tumor growth of Caco-2 cells in nude mice (*n* = 5).

To examine the combined effect of 4D3 and an anti-EGFR antibody (C225), spheres of Caco-2 cells were treated with C225 with 4D3 or anti-mouse CLDN4 antibody, which does not recognize human CLDN4. Concurrent treatment with C225 and 4D3 resulted in more effective inhibition of sphere formation than treatment with C225 alone (Figure 5A). Fluorescence-labeled C225 was localized on the cytoplasmic membrane at the sphere surface, as well as inside the sphere following co-treatment with 4D3, whereas C225 was restricted to the surface of the sphere when used alone (Figure 5B). In sphere culture, co-treatment with C225 and 4D3 inhibited EGFR phosphorylation as effective as knockdown of EGFR (Figure 5C). Co-treatment with C225 and 4D3 also decreased expression of nucleostemin (NS), a stem cell marker. Notably, 4D3 alone provided a modest decrease of NS.

In a nude mouse subcutaneous tumor model, concurrent treatment with C225 and 4D3 resulted in more effective inhibition of tumor growth than C225 alone in both Caco-2 (*ras* wild type) and HT29 (*ras* wild type, *Braf* mutant) cells (Figure 5D, 5E), both of which are reported to respond to C225 [20]. We next attempted the sequential treatment of 4D3 followed by C225.

The effect of the time interval between 4D3 treatment and C225 treatment on the phosphorylation of EGFR was examined. Phosphorylation levels of EGFR were decreased in a time interval-dependent manner (Figure 5F). Finally, the sequential treatment of 4D3 followed by C225 was examined in an animal model. Sequential treatment with a 6-h time interval provided a more effective inhibitory effect on tumor growth than concurrent treatment (Figure 5G).

## DISCUSSION

CLDN4 is a dominant tight junction protein in the colonic epithelium that functions in cooperation with CLDN3 and CLDN1. Their homotypic binding regulates basolateral material flow in the membrane. Dysregulation of this function contributes to the initiation and progression of cancer [5]. Altered CLDN expression has been reported in many types of epithelial cancers in a stage- and tumor-specific manner [5, 21]. Here, we found that CLDN4 is expressed in colonic adenomas and adenocarcinomas. CLDN4 expression correlated with pathological stage and distant metastasis; however, it was inversely correlated with local progression. In contrast, bladder cancer shows a clear correlation between CLDN4 expression and parameters associated with cancer progression [11].

One reason for the weaker correlations between CLDN4 expression and clinicopathological parameters in this study may be the influence of inflammatory cytokines. For example, other inflammatory cytokines (such as, as shown here, TNFα) also affect CLDN expression. In the colonic environment, the bacterial flora is a key modulator of immune responses, such as cytokine production, in the host mucosa. In addition to immune cells, epithelial cells also produce cytokines. Among the gut flora, *C. perfringens* plays a specific role in the colonic epithelium, producing CPE, which binds avidly to CLDN3 and CLDN4. This binding disrupts tight junctions and injures the cytoplasmic membrane, and this activity underlies the pathogenicity of *C. perfringens* during dysenteric colitis.

Inflammatory cytokines induce tight junction remodeling; however, their effects on claudin protein levels depend on types of cytokines and cells [22, 23]. We confirmed the downregulation of CLDN4 by TNFα, whereas interferon-gamma increases CLDN7 expression [22]. TNFα decreased CLDN3 and CLDN4 proteins; however, increased CLDN1 and CLDN7 proteins, which suggests that TNFα decreased CLDN4 but not impair the tight junction function by compensation with other claudins. This phenomenon might affect the relationship between CLDN4 expression and tumor progression (pT). Further examination is necessary for cytokine-induced tight junction remodeling; however, TNFα might not be suitable for increasing permeability of anti-cancer drugs.

Our data showed that CPE induced TNFα production in HT29 cells and that this was associated with the suppression of CLDN4 expression. Interestingly, CPE upregulated *CLDN4* mRNA levels in the absence of TNFα, suggesting that TNFα counteracts some of the direct effects of CPE on *CLDN4* transcription. In some types of malignancies, an intrinsic decrease in CLDN4 expression has been associated with the loss of cancer cell adherence, invasion, and metastasis [24, 25]. In contrast, increased CLDN4 expression is associated with metastasis in some types of malignancies [11]. Our current data showed that CLDN4 expression in the metastatic foci was significantly higher than that in the primary tumors. This may be because *C. perfringens* and TNFα have more moderate effects than those elicited by the colonic environment. Reduced CLDN4 expression has been associated with epithelial–mesenchymal transition (EMT) [26]. However, it is premature to speculate that CLDN4 is a biomarker for EMT or metastasis, since many factors can modulate its expression.

Tight junctions are borders between the lumen and the internal environment of the epithelium [4]. The borders play bidirectional roles, acting as both fences and barriers [27, 28]. The fence function involves the retention of growth factors or other tumor-associated factors inside the tumor. The 4D3 anti-CLDN4 extracellular domain antibody abrogated this fence function, thus releasing EGF and implied hypoxic condition; in turn, this reduced EGFR phosphorylation and inhibited cell growth. This perturbation of the fence function was more pronounced in spheres than in monolayer cultures, most likely due to the increased size of the internal environment in spheres. Importantly, 4D3-induced impairment of fence function decreased stem cell phenotype. EGFR activation is associated with nucleostemin expression via wnt signal [29]. The finding suggests that leakage of EGF from tumor microenvironment by 4D3 might result inhibition of stem cell phenotype of cancer cells.

The barrier function prevents external substances such as anticancer drugs from entering cells. Thus, treatment with 4D3 rendered cells more permeable to 5-FU, increasing its intracellular concentration in the tumor environment. This is similar to the enhancement in anticancer drug concentrations in tumor tissue that is elicited by increasing vascular permeability [30]. Similarly, 4D3 has been shown to increase drug permeability in a bladder cancer model [11]. Thus, CLDN4 targeting provides antitumoral effects by damaging both the fence and barrier functions of tight junctions. Our data showed that 0.5 μg/ml of 4D3 doubled intracellular 5-FU concentration, which means twice effect is expected by the same dosage. The data suggest the possibility to overcome 5-FU resistance by concurrent administration of 4D3 and 5-FU.

Our previous study showed that CLDN4 regulates the permeability of tumor cells both *in vitro* and *in vivo* [11]. However, in tumor tissues, the functional maturity of the tight junction is controversial. Tumor tissues lack the polarity found in the mature mucosa. The tight junctions in tumor tissues may therefore be partially functional and redundant in comparison with those in the normal mucosa. Recent studies have elucidated non-tight junction roles for CLDNs, such as their ability to regulate signal transduction [31, 32]. Whether 4D3 regulates these newly described activities of CLDN4 requires further investigation.

In CRC treatment, molecular targeting by antibodies is becoming standard therapy [33]. We examined the effect of 4D3 on the antitumoral activity of the anti-EGFR antibody C225, known as cetucimab, which is commonly used to treat CRC. Considering the molecular sizes of antibodies, it is thought to be a problem that destruction of tight junction by 4D3 might be enough to permeation of another antibody [11, 34, 35]. To test the effect of a combination of these antibodies, we used a sphere assay, which provides a three-dimensional structure mimicking a tumor environment [36]. These antibodies permeated the spheres following concurrent treatment, whereas in the absence of 4D3, the C225 antibody accessed the sphere surface only. The effect of 4D3 on the inhibitory activity of C225 was maximized by sequential treatment of the two antibodies. This suggests that the impairment of tight junctions by 4D3 increases with longer incubation times. In nude mouse tumors, the increased effectiveness of a 6-h interval between 4D3 and C225 treatment was confirmed.

CLDN4 is thought to be a promising therapeutic target for drug development [18, 19]. Through *in vivo* models, we observed that combinations of 4D3 with 5-FU or C225 exhibited synergistic effects, resulting in increased efficacy over that of single-agent treatments. The suppression of CLDN4 expression using TNFα showed that this synergy was inversely correlated with CLDN4 expression. These findings suggest that 4D3 is a strong candidate antitumoral agent for the treatment of CRC cells expressing CLDN4, especially if used in combination with other anticancer drugs.

## MATERIALS AND METHODS

### Surgical specimens

We reviewed the pathological diagnosis and clinical data of 124 patients with CRC diagnosed in the Department of Molecular Pathology, Nara Medical University from 2012 to 2015. Basic patient information is summarized in Table 1. As written informed consent was not obtained, any identifying information was removed from the samples prior to analysis, in order to ensure strict privacy protection (unlinkable anonymization).

Fresh tumor tissues were obtained from in 11 CRCs, which were all pT3 cases. Tissues were frozen and stored in –80°C. Whole lysates were extracted from the tissues as previously described [37]. Whole lysates were subjected to ELISA.

All procedures were performed in accordance with the Ethical Guidelines for Human Genome/Gene Research enacted by the Japanese Government and were approved by the Ethics Committee of Nara Medical University (Approval Number 937).

### Cell lines

HT29 and Caco-2 human colon cancer cell lines were purchased from Dainihon Pharmaceutical Co. (Tokyo, Japan). Cells were cultured in Dulbecco's modified Eagle's medium (DMEM) supplemented with 10% fetal bovine serum (FBS) at 37°C in 5% CO_2_. Cell growth was assessed using a tetrazolium (MTT) dye assay, as previously described [38].

### Antibody and reagents

The anti-human CLDN4 extracellular domain antibody 4D3 was developed by immunizing rats with a plasmid vector encoding human CLDN4 [11]. Human tumor necrosis factor (TNF)-α (Serotec, Hercules, CA, USA), anti-human epithelial growth factor receptor (EGFR) antibody (clone C225, cetuximab; Funakoshi Co., Ltd., Tokyo, Japan), CPE (Sigma, St. Louis, MO, USA), and 5-FU (Wako Pure Chemical Corp. Ltd., Osaka, Japan) were purchased commercially.

### Sphere assay

Caco-2 cells (5 × 10^4^) were grown in stem cell medium (Sigma) in 6-well bacteriological grade plates (Gibco, Grand Island, NY, USA) and incubated at 37°C in 5% CO_2_. After 5 h, cells were treated with 5-FU and/or 4D3 for 24 h.

### Animals

BALB/c nude mice (4 weeks old, male) were purchased from SLC Japan (Shizuoka, Japan). The mice were maintained according to the institutional guidelines approved by the Committee for Animal Experimentation of Nara Medical University, in accordance with the current regulations and standards of the Ministry of Health, Labor, and Welfare.

To establish a subcutaneous tumor model, Caco-2 and HT29 cells (1 × 10^7^) were inoculated subcutaneously into the scapular tissues of nude mice. Then, with five mice in each group, 5-FU (10 mg/kg body weight) and/or 4D3 (1 mg/kg body weight, diluted with saline) were injected into the peritoneal cavity simultaneously on days 1, 3, and 7. Tumor size was monitored weekly.

For the establishment of liver and peritoneum metastasis models, HT29 cancer cells (1 × 10^6^) were inoculated into the spleen and peritoneal cavity, respectively. With five mice in each group, 5-FU (3 mg/kg body weight) and/or 4D3 (0.2 mg/kg body weight; [11]) were injected into the peritoneal cavity simultaneously on days 7, 14, and 21. Mouse livers were observed for 4 weeks following inoculation. The livers were sectioned into 2-mm-thick slices, and metastatic foci were counted using a stereomicroscope (Nikon, Tokyo, Japan). The numbers of metastatic foci in the peritoneal cavity were counted macroscopically.

In addition, Caco-2 cells (1 × 10^7^) were inoculated subcutaneously into the scapular tissues of nude mice. With five mice in each group, 4D3 (1 mg/kg body weight, diluted with saline) and/or C225 antibody (3 mg/kg body weight, diluted with saline) were injected into the peritoneal cavity once a week for 4 weeks. Tumor size was monitored weekly.

### Immunohistochemistry

Consecutive 4-μm sections were immunohistochemically stained using 0.2 μg/mL 4D3 and a previously described immunoperoxidase technique [39]. Secondary antibodies (Medical and Biological Laboratories, Nagoya, Japan) were used at a concentration of 0.2 μg/mL. Tissue sections were color-developed with diamine benzidine hydrochloride (DAKO, Glastrup, Denmark) and counterstained with Meyer's hematoxylin (Sigma). We counted immunopositive cells at the cytoplasmic membrane. Staining strength was scored from 0 to 3 (a score of 1 was used to describe the expression level in normal colonic epithelium). The staining index was calculated as the staining strength score multiplied by the staining area (%), and the resulting scores were defined as follows: none (index, 0), weak (index, 1–100), medium (index, 101–200), and high (index, 201–300). For a negative control, non-immunized rat IgG (Santa-Cruz Biotechnology, Santa-Cruz, CA, USA) was used as a primary antibody.

For immunostaining of spheres, spheres were treated with 4D3 and C225 antibodies labeled with HiLyte Fluor 555 and 647 (Dojindo, Kumamoto, Japan), respectively, for 4 h in accordance with the manufacturer's instructions. The spheres were observed by an all-in-one florescence microscope (Keyence Corp., Osaka, Japan).

### Immunoblot analysis

Whole-cell lysates were prepared as previously described [37]. Lysates (20 μg) were subjected to immunoblot analysis using SDS-PAGE (12.5%), followed by electrotransfer onto nitrocellulose filters. The filters were incubated with primary antibodies, followed by peroxidase-conjugated IgG antibodies (Medical and Biological Laboratories). Anti-tubulin antibody was used to assess the levels of protein loaded per lane (Oncogene Research Products, Cambridge, MA, USA). The immune complex was visualized using an Enhanced Chemiluminescence Western-blot detection system (Amersham, Aylesbury, UK). Antibodies for CLDN4 (4D3), and TNFα, EGFR, CLDN1, CLDN3, CLDN7, hypoxia inducible factor (HIF)-1α, nucleostemin (NS) (Abcam plc., Cambridge, UK) and phsophorylated EGFR (Santa Cruz Biotechnology) were used as primary antibodies.

### Reverse transcription-polymerase chain reaction

To assess human *CLDN4* mRNA expression, reverse transcription-polymerase chain reaction (RT-PCR) was performed with 0.5 μg total RNA extracted using an RNeasy kit (Qiagen, Germantown, MD, USA). The primer sets for human *CLDN4* amplification were as follows: forward, 5′-CTC CAT GGG GCT ACA GGT AA-3′ and reverse, 5′-AGC AGC GAG TCG TAC ACC TT-3′ (NCBI Reference Sequence: NM_001305.4; synthesized by Sigma Genosys, Ishikari, Japan). PCR products were electrophoresed in a 2% agarose gel and stained with ethidium bromide. β-Actin (*ACTB*) mRNA was also amplified for use as an internal control (GenBank Accession No. NM_001101).

### Enzyme-linked immunosorbent assay

Enzyme-linked immunosorbent assay (ELISA) kits were used to measure the concentrations of EGFR, phosphorylated EGFR, human TNFα (R&D Systems Inc., Minneapolis, MN, USA), CPE (TECHLAB, Blacksburg, VA, USA), HIF-1α (Cell Biolabs, Inc., San Diego, CA, USA), human CLDN4 (Cusabio Biotech, Wuhan, China), and 5-FU (anti-5-Fluorouracil antibody-derived ELISA, MABEL Inc., Kyoto, Japan), according to the manufacturers’ instructions.

### Short interfering RNA assay

FlexiTube short interfering RNAs (siRNA) targeting *TNFA* and *EGFR* were purchased from Santa Cruz Biotechnology. AllStars Negative Control siRNA (Qiagen) was used as a control. Cells were transfected with 50 nM siRNA using Lipofectamine 2000 (Invitrogen, Carlsbad, CA, USA), according to the manufacturer's instructions.

### Statistical analysis

Statistical significance was calculated using two-tailed Fisher's exact, chi-square, and unpaired Mann-Whitney tests with InStat software (GraphPad, Los Angeles, CA, USA). Statistical significance was defined as a two-sided *p*-value of < 0.05.

## Abbreviations

CLDN: claudin
CRC: colorectal cancer
5-FU: 5-fluorouracil
EGFR: epithelial growth factor receptor
CPE: *Clostridium perfringens* enterotoxin
TNF: tumor necrosis factor
HIF: hypoxia inducible factor
NS: nucleostemin
EMT: epithelial–mesenchymal transition.
